# Food Quality Assessed by Chemometrics

**DOI:** 10.3390/foods9070897

**Published:** 2020-07-08

**Authors:** Christelle M. Andre, Christos Soukoulis

**Affiliations:** 1 The New Zealand Institute for Plant and Food Research Limited (PFR), Private Bag 92169, Auckland 1142, New Zealand; christelle.andre@plantandfood.co.nz; 2 Systems and Bioprocessing Engineering group, Environmental Research and Innovation (ERIN) Department, Luxembourg Institute of Science and Technology (LIST), 5 avenue des Hauts Fourneaux, L4422 Esch-sur-Alzette, Luxembourg

**Keywords:** food quality, chemometrics, food authenticity, process optimization and modelling, foodomics, data mining, food product development

## Abstract

Food market globalization, food security as well as increasing consumer demand for safe, minimally processed and healthy food impose the need to establish new approaches for identifying and assessing food quality markers. Nowadays, food industry stakeholders are challenged to assure food quality and safety without compromising several prerequisites such as sustainable and ecologically resilient food production, prolonged shelf life, satisfactory sensory quality, enhanced nutritional value and health-promoting properties. In addition, food fraud related to deliberate product mislabeling or economically intended adulteration is of major concern for both industry and regulatory authorities due to cost and public health implications. Notwithstanding the great number of state-of-the-art analytical tools available for quantifying food quality markers, their implementation results in highly complex and big datasets, which are not easily interpretable. In this context, chemometrics e.g., supervised and unsupervised multivariate exploratory analyses, design-of-experiment methodology, univariate or multivariate regression modelling etc., are commonly implemented as part of food process optimization and food quality assessment. In this Special Issue, we aimed to publish innovative research and perspective papers on chemometric-assisted case studies relating to food quality assessment, food authenticity, mathematical modelling and optimization of processes involved in food manufacturing.

## General Remarks

The advancement in the fields of nutrigenomics, food biochemistry, analytical chemistry, food processing technology as well as consumer and sensory science has significantly advanced the knowledge of food systems. In addition, the evolution of instrumental analysis tools such as chromatographic, spectroscopic and spectrometric methods, metabolomics, proteomics and genomics has substantially improved our capability in unveiling and understanding the complexity of food matrices in terms of composition, biophysics, microstructure, sensorial cross-modality and biological activity [1]. In food research and development (R and D) applications, the association of a single independent parameter (e.g., compositional, processing or storage conditions, phenotype, genotype, geographical origin etc.) to a single food matrix property (physicochemical, rheological, textural, sensory, olfactory, bio-functional marker etc.) is considered as a standard experimental design (bottom-up) approach [2,3]. In spite of its simplicity, the “bottom-up” experimental design approach provides a fast-tracking tool for understanding the major mechanisms underlying in food matrix structural conformation, techno-functionality, biological activity and sensory cross-modality [1]. On the other hand, the bottom-up experimental design is per se restricted when multicausal non-linear interactions between extrinsic and intrinsic characteristics of the food matrix need to be understood [2]. In this context, a holistic characterization of food systems, i.e., food matrix, food processing conditions, cross-modal sensoriality, physiological effects etc., is more informative and integrative for R and D applications; yet, limitations associated with the production of complex and large datasets that can be poorly validated and interpreted using exclusively univariate statistical methods are generally recognized. Chemometrics is considered as a rapid and efficient way to analyze a food matrix beyond univariate dimensionality and unveil the hidden information in complex instrumental datasets [1].

The term chemometrics is used to denote “the chemical discipline that uses mathematical and statistical methods to (a) design or select optimal measurement procedures and experiments and (b) provide maximum chemical information by analyzing chemical data” [4]. With regard to food research and innovation, chemometrics can be employed to

(a) facilitate the exploration and interpretation of chemical and biological information acquired via instrumental analysis;
(b) identify chemical, biochemical or biological markers associated with food quality and safety;
(c) identify the authenticity, quality, bio-functional and nutritional aspects of food on the basis of geographical and botanical origin, presence of non-disclosed food additives or contaminants;
(d) construct robust mathematical models for monitoring food process unit operations and predict shelf-life of processed food;
(e) understand the interactions between sensory modalities and compositional, physical, textural and microstructural properties of food;
(f) optimize the formulation and processing conditions in R and D-associated problem-solving applications.

In the present Special Issue, one perspective paper [3] and four chemometric-driven food product application case studies [5,6,7,8] were published. In their paper, Truong and co-workers [3] provided a concise overview of the key issues associated with the implementation of chemometrics in food research and development. In general, food research data may be either qualitative (e.g., nominal, dichotomous and ordinal) or quantitative (e.g., continuous scales of experimental parameters such as concentration, mass, volume, time, pressure, temperature etc., intervals or ratios) [9]. Three major chemometric approaches are usually applied in addressing food R and D questions including exploratory (unsupervised statistic methods such as Principal Component Analysis and Cluster Analysis), classification (supervised statistic methods such as Discriminant Analysis e.g., LDA, PLS-DA etc., K-nearest neighbors, SIMCA and Artificial Neural Networks), regression and calibration (Multiple Linear Regression, Partial Least Squares, Principal Component Regression) [3,9]. Despite the growing popularity of the use of chemometrics in food R and D applications, the lack of understanding of chemometric tools, the absence of Design of Experiments (DoE) or the use of ill-designed DoE, as well as inconsistencies related to model calibration, validation and update are recognized as common cases of chemometrics misuse [3]. In this context, good practices in chemometric-assisted data analysis and interpretation should meet several criteria [3]:

(a) good knowledge of the food analysis methodology (instrumental, analytical, sensory, structural, textural etc.) and its inherent validation characteristics (e.g., accuracy, precision, specificity, detection limited, quantitation limit, linearity, robustness etc.);
(b) sufficient understanding of the food system (e.g., inherent physicochemical, compositional, structure conformational and textural characteristics);
(c) robust design of experiments;
(d) good understanding of the interplay between sample, sampling procedure and analytical data acquisition.

To bridge the gap between theory and practice, selected case studies in the domain of food quality assessment by chemometrics were included in the specific Special Issue. Sousa and co-workers [5], evaluated the feasibility of Fourier Transform Infrared Spectroscopy (FTIR), coupled with exploratory (PCA) and calibration/modelling (PLS-R) statistical tools, in detecting adulteration of Atlantic salmon (*Salmo salar*) with salmon trout (*Oncorhynchus mykiss*) muscle. PCA was successful in clustering the muscle food sample on the basis of the adulteration level without detecting any effect of the storage time (i.e., at 3 °C for 0, 72, 160 and 240 h). The absorbance bands corresponding to 721 (–HC=CH–, –CH_2_–), 1097 (ester of –C–O–group), 1370 (CH_3_ group), 1464 (CH_3_ and CH_2_), 1665 (–C=C–), 2850 to 2925 (symmetrical and asymmetric methylene) and 3009 cm^−1^ (=C–H). PLS regression analysis using leave-one-out (LOO) cross validation allowed the discrimination of Atlantic salmon-based muscle food adulterated with salmon trout regardless of the chilling storage time and adulteration level. In a following study, Fengou and co-workers [6] investigated the ability of spectroscopy (FTIR, visible spectroscopy (VIS) and multispectral image (MSI) acquisition)-based sensors to predict (using PLSR based models) the microbiological spoilage in minced pork under isothermal and dynamic temperature (4, 8 and 12 °C) storage conditions. FTIR-based sensors exerted the best microbiological spoilage predicting power followed by MSI and VIS sensors. This was mainly ascribed to the fingerprinting of the spectral range of 1800–900 cm^−1^, which was associated with the production of amides and amines via either enzymatic (autolysis) or microbiological induced proteolysis. On the other hand, VIS and MSI provided important information regarding the moisture and protein content of the minced meat samples. When fused sensor exemplars were used (FTIR/VIS/MSI), the overall predictive power of the obtained model was higher than that of the individual VIS or MSI, yet lower than that of the individual FTIR.

A geographical origin (different production sites) classification study of green coffee beans using Near Infrared Spectrometry (NIR) was showcased by Okubo and Kurata [7]. The flat surface of the coffee beans was scanned by means of NIR and the obtained spectral data were analyzed by Soft Independent Modelling of Class Analogy (SIMCA). The spectral region 1850–1950 cm^−1^ ascribed to C=O, H_2_O, caffeine, chlorogenic acid, protein and lipids and carbohydrates provided the most important information for discriminating the coffee bean samples in terms of geographical origin. An overall classification percentage of 73% was achieved with the samples obtained from Red Sea closing countries (Ethiopia and Yemen) to be almost completely (93% and 100%, respectively) discriminated from the Tazmania, Indonesia and Cuba analogues. 

In their recent study, Tsevdou and co-workers [8] used univariate regression and exploratory multivariate analysis tools to investigate the impact of milk pre-processing parameters (high pressure vs. heat-treated and transglutaminase-assisted milk protein cross-linking) on the quality and bio-functional characteristics of yoghurt. PCA revealed that the high-pressure pre-processing of milk (ovine and bovine) enhanced the angiotensin converting enzyme (ACE) inhibitory activity throughout the foreseen shelf life of yoghurt. Pre-treatment of milk with transglutaminase favored the expression of anti-inflammatory genes (i.e., TGFB1) and hampered the expression of pro-inflammatory genes such as IL1B and IL12B from monocytes. Ovine-based yoghurts exerted substantially better anti-hypertensive and immunomodulatory properties than the bovine-based exemplars.

## References

[1] Roberts J.J., Cozzolino D. (2016). An Overview on the Application of Chemometrics in Food Science and Technology—An Approach to Quantitative Data Analysis. Food Anal. Methods.

[2] Fardet A. (2014). New Approaches to Studying the Potential Health Benefits of Cereals: From Reductionism to Holism. Cereal Foods World.

[3] Truong V.K., Dupont M., Elbourne A., Gangadoo S., Rajapaksha Pathirannahalage P., Cheeseman S., Chapman J., Cozzolino D. (2019). From Academia to Reality Check: A Theoretical Framework on the Use of Chemometric in Food Sciences. Foods.

[4] Forina M., Casale M., Oliveri P., Brown S.D., Tauler R., Walczak B. (2009). 4.04-Application of Chemometrics to Food Chemistry. Comprehensive Chemometrics.

[5] Sousa N., Moreira M.J., Saraiva C., De Almeida J.M.M.M. (2018). Applying Fourier Transform Mid Infrared Spectroscopy to Detect the Adulteration of Salmo salar with Oncorhynchus mykiss. Foods.

[6] Fengou L.-C., Spyrelli E., Lianou A., Tsakanikas P., Panagou E.Z., Nychas G.-J.E. (2019). Estimation of Minced Pork Microbiological Spoilage through Fourier Transform Infrared and Visible Spectroscopy and Multispectral Vision Technology. Foods.

[7] Okubo N., Kurata Y. (2019). Nondestructive Classification Analysis of Green Coffee Beans by Using Near-Infrared Spectroscopy. Foods.

[8] Tsevdou M., Theodorou G., Pantelaiou S., Chatzigeorgiou A., Politis I., Taoukis P. (2020). Impact of Type and Enzymatic/High Pressure Treatment of Milk on the Quality and Bio-Functional Profile of Yoghurt. Foods.

[9] Granato D., Putnik P., Kovačević D.B., Santos J.S., Calado V., Rocha R.S., Cruz A.G.D., Jarvis B., Rodionova O.Y., Pomerantsev A. (2018). Trends in Chemometrics: Food Authentication, Microbiology, and Effects of Processing. Compr. Rev. Food Sci. Food Saf..

